# Sage Essential Oil as an Antimicrobial Agent against *Salmonella enterica* during Beef Sous Vide Storage

**DOI:** 10.3390/foods12224172

**Published:** 2023-11-19

**Authors:** Robert Gál, Natália Čmiková, Miroslava Kačániová, Pavel Mokrejš

**Affiliations:** 1 Department of Food Technology, Faculty of Technology, Tomas Bata University in Zlín, Vavrečkova 5669, 760 01 Zlín, Czech Republic; gal@ft.utb.cz; 2 Institute of Horticulture, Faculty of Horticulture and Landscape Engineering, Slovak University of Agriculture, Tr. A. Hlinku 2, 94976 Nitra, Slovakia; n.cmikova@gmail.com; 3 School of Medical & Health Sciences, University of Economics and Human Sciences in Warsaw, Okopowa 59, 01 043 Warszawa, Poland; 4 Department of Polymer Engineering, Faculty of Technology, Tomas Bata University in Zlín, Vavrečkova 5669, 760 01 Zlín, Czech Republic; mokrejs@utb.cz

**Keywords:** sage essential oil, beef tenderloin, stabilization, safety, under vacuum, foodborne pathogen, novel application, active substance

## Abstract

Sous-vide is a process comprising vacuum-sealing food, heating it to the desired temperature, and circulating it in a water bath in a sous vide machine. This cooking technique is increasingly common in homes and catering establishments due to its simplicity and affordability. However, manufacturers and chef’s recommendations for low-temperature and long-term sous-vide cooking in media raise food safety concerns, particularly when preparing beef tenderloin. In this study, *Salmonella enterica* was found to be inactivated by heat and sage essential oil (EO) in beef samples from *musculus psoas major* that had been sous vide processed. To determine whether heat treatment was likely to increase the sous vide efficiency, *S*. *enterica* and sage EO were mixed. After being vacuum-packed and injected with *S. enterica*, the samples were cooked at 50–65 °C through the sous vide technique for the prescribed time. On days 1, 3, and 6, the amounts of *S. enterica*, total bacteria, and coliform bacteria were measured in the control and treated groups of beef processed sous vide. Mass spectrometry was used to identify bacterial isolates on different days. On each day that was measured, a higher number of all the microbiota was found in the samples exposed to 50 °C for 5 min. The most frequently isolated microorganisms from both groups of samples were *Pseudomonas fragi* (17%), *Pseudomonas cedrina* (8%), and *Proteus vulgaris* (8%); in the treated group, also *S. enterica* (21%), *Pseudomonas fragi* (13%), and *Pseudomonas veronii* (6%). After the heat treatment of samples at 65 °C for 20 min, the total count of bacteria and coliform bacteria was zero. It has been shown that adding sage essential oil (EO) in combination with sous vide processing technique leads to the stabilization and safety of beef tenderloin.

## 1. Introduction

The cooking technique known as sous vide, or “under vacuum”, involves vacuum-sealing uncooked food and heating it in a temperature-controlled hot water bath [1]. Contrary to other cooking techniques, sous vide uses uniform heat conduction while the food is submerged to achieve a precise level of cooking throughout the result. Due to the availability of numerous cookers that are accessible, user-friendly, and reasonably priced, sous vide cooking has grown significantly in popularity over the past ten years [2].

*Salmonella enterica* and other dangerous germs are occasionally present in raw meat. Combining information from numerous surveys, it was discovered that 3.8% of raw (mainly minced) beef and 1.3% of cold beef carcasses were contaminated with *Salmonella* [3]. Controlling these infections is crucial for ensuring food safety, especially regarding raw products with long shelf lives, like the marinated meats mentioned by Kargiotou et al. [4]. Adequate chilling inhibits the growth of *Salmonella* [5]. Between 80% and 90% of salmonellosis cases in industrialized nations are linked to consuming foods with animal products [6,7]. The gastrointestinal tract of animals is known to be colonized by *Salmonella* without any clinical or pathologic-anatomic symptoms [7,8,9]. As a result, during slaughter, carcasses may become tainted with *Salmonella*. The primary means of transmission for this foodborne disease are contaminated raw or undercooked red meats. Although official meat inspection is conducted correctly in the slaughterhouse, it is likely that *Salmonella* is frequently not found on the surface of (and deeper inside) the corpses [9].

During their manufacturing, sale, and distribution, raw and/or processed foods are vulnerable to contamination [10]. As a result, preservatives are currently required in order to stop the spread of food deterioration bacteria in the food sector [11]. Although a few commercially available food preservatives contain essential oils (EOs), relatively few investigations of the activity of EOs in foods had been published before the early 1990s [12]. In general, lowering the pH level of food, increasing the temperature at which food is stored, and increasing the O_2_ content of the packing all increase bacteria’s susceptibility to the antibacterial effect of EOs. The physical structure of food may limit the antibacterial activity of EO. Furthermore, it has been demonstrated that several EOs are superior bactericidal agents compared to the widely applied preservatives for meat applications [13].

The aims of this study were (i) to determine the antimicrobial activities of EO extracted from *Salvia officinalis*; (ii) to assess the efficacy of these EO as antimicrobial after their conservation at 4 ± 2 °C for 6 days; (iii) to test the antibacterial activity of these EO against foodborne pathogens belonging to *Salmonella* genus, inoculated in sous vide beef meat.

## 2. Materials and Methods

### 2.1. Inoculum Preparation

*Salmonella enterica* subsp. *enterica* CCM 4420 was used for the experiment. The microbial inoculum has been cultured for 24 h on Mueller Hinton agar (MHA, Oxoid, Basingstoke, UK) at 37 °C. Adjustment of the inoculum to optical density 0.5 McFarland standard (1.5 × 10^8^ CFU/mL) followed; and 100 μL was added to the meat samples.

### 2.2. Essential Oil

*Salvia officinalis* EO (sage, SOEO), which was created by steam distilling dry top, was obtained from Hanus s.r.o. in Nitra, Slovakia. For the duration of the analyses, it was kept at 4 ± 2 °C in the dark. The main components in *S. officinalis* EO were identified using gas chromatography/mass spectrometry (GC/MS) and gas chromatography (GC-FID). The chemical composition comprised α-thujone at 24.6%, camphor at 20.6%, 1,8-cineole at 12.1%, and α-humulene [14].

### 2.3. Sample of Beef Meat Preparation

Beef thigh meat samples (*muscles psoas major*) belonging to the Charolais animal breed used in this experiment were purchased from the certified merchant (Steinhauser, Ltd., Tišnov, the Czech Republic). The meat samples were transported in less than 120 min under standardized conditions to ensure safety and hygiene to the microbiological department and stored at the temperature of 4 ± 2 °C prior to the analyses. The meat underwent dicing, and 5 g samples were subjected to a solution of *Salvia officinalis* essential oil (EO). A 2.0% (*w*/*w*) solution of *Salvia officinalis* EO was created by dissolving it in sunflower oil through mixing at 25 °C for 10 min. Meat samples were immersed in the *Salvia officinalis* EO solution for 10 s. Following the extraction of the meat samples from the coating solution, the surplus solution was permitted to drip for 30 s; subsequently, the meat samples were vacuum-sealed using a vacuum packer from Concept (Choce, Czech Republic). Premium sunflower oil was procured from an authorized supplier [15]. A total of 480 diverse beef samples were examined in the analysis. In our experiment, the samples were provided in the way described below:

BM: Beef meat samples vacuum-packed in PE bags and kept at 4 ± 2 °C for anaerobic storage before being heated to 50–65 °C for 5–20 min.

BMSEEO: Beef meat samples vacuum-packed in PE bags, treated with *S. enterica* and 2% sage EO, and kept anaerobically at 4 ± 2 °C for 5 to 20 min.

Raw uncooked beef was used to prepare the control samples on day 0. The samples were given the essential oil, and maceration was carried out for 24 h on them. The CASO SV1000 sous vide machine was used to cook the samples.

### 2.4. Cultivation of the Samples

Microbiological analysis was conducted at 4 ± 2 °C on days 1, 3, and 6. A temperature of 4 ± 2 °C was chosen as the threshold temperature that is more favorable for the survival of the food-borne pathogen *Salmonella enterica*. First, 45 mL sterile saline solution of a 0.1% concentration was used to dilute the 5 g samples in an Erlenmeyer flask. Then, homogenization of the samples for 30 min in the shaking device (Burgwedel, Germany, GFL 3031) followed. The following microbial species were assessed: Coliforms were detected in the bacterial culture medium Violet Red Bile Lactose Agar (VRBL, Oxoid, Basingstoke, UK), with an incubation period at 37 °C for 24 to 48 h. Total viable counts (TVC) were cultivated on Plate Count Agar (PCA; Oxoid, Basingstoke, UK) and incubated at 30 °C for 48–72 h. Xylose Lysine Deoxycholate Agar (XLD; Oxoid, Basingstoke, UK), underwent incubation at 37 °C for 24–48 h. Subsequently, eight colonies per Petri dish were briefly re-inoculated on Trypton Soya Agar (TSA; Oxoid, Basingstoke, UK) for 24 h.

### 2.5. Identification of Bacteria with Mass Spectrometry

The MALDI-TOF MS Biotyper (Matrix-Assisted Laser Desorption/Ionization Time of Flight) from Bruker Daltonics in Bremen, Germany, equipped with reference libraries, was employed for the identification of microorganisms isolated from beef tenderloin samples [15].

### 2.6. MALDI-TOF Matrix Solution Preparation

A stock solution was produced, and it became an organic material. The standard solution comprised 2.5% CF_3_COOH, 47.5%H_2_O, and 50% CH_3_CN. To create a 1 mL stock solution, a mixture of 500 mL of 100% pure CH_3_CN, 475 mL of purified H_2_O, and 25 mL of 100% pure trifluoroacetic acid was combined. The organic solvent, along with the portioned “HCCA matrix,” was prepared and mixed in a 250 mL Eppendorf flask [15]. The ingredients for the matrix were delivered by Lambda Life in Bratislava, Slovakia.

### 2.7. Identification of Microorganisms

Samples were generated in accordance with the previously outlined instructions [15]. Eight distinct colonies were chosen from the Petri dish. The biological material was transferred from the Petri dish to an Eppendorf flask containing 300 mL of distilled water, stirred, and supplemented with 900 mL of ethanol. Subsequently, the mixture underwent centrifugation using a ROTOFIX 32A, manufactured by Ites in Vranov, Slovakia, for two minutes at 10,000× *g*. Following the removal of the supernatant, the precipitate was isolated from the Eppendorf tube and left to air-dry at laboratory temperature (20 °C). Subsequently, 30 mL of 70% formic acid and 30 mL of acetonitrile were administered to the particle. The resulting mixture underwent centrifugation at 10,000× *g* for 2 min. One milliliter of the liquid obtained was utilized to coat a MALDI plate, followed by the addition of one milliliter of MALDI matrix solution. The specimens were desiccated before undergoing analysis in a MALDI-TOF mass spectrometer (Bruker, Daltonics, Bremen, Germany) to ascertain the identification of microorganisms. Mass spectra were automatically generated utilizing the microflex LT MALDI-TOF mass spectrometer (Bruker Daltonics, Bremen, Germany), configured to operate in the linear positive mode within a mass range of 2000–20,000 Da. Calibration of the instrument was performed using the Bruker bacterial test standard. The results obtained from the mass spectra were scrutinized using the MALDI Biotyper 3.0 software (Bruker Daltonics, Bremen, Germany). Identification criteria encompassed the following: scores within the range of 2.300–3.000 indicated highly probable species identification at the genus level; 2.000–2.299 signified genus identification with less probable species identification; 1.700–1.999 indicated probable genus identification; and a score below 1.700 was considered an unreliable identification [15].

### 2.8. Statistical Evaluations

Each analysis and test was conducted in triplicate. Microsoft Excel was utilized to compute the mean and standard deviation (SD) of microbial counts. A one-way analysis of variance (ANOVA) was carried out using Prism 8.0.1 (GraphPad Software, San Diego, CA, USA) with a significance level of 0.05 before applying Tukey’s test. Data analysis was performed using the SAS^®^ software version 8 (SAS Institute, North Carolina, USA) [15].

## 3. Results

### 3.1. Number of Bacteria in log CFU/g

**Figure 1:**
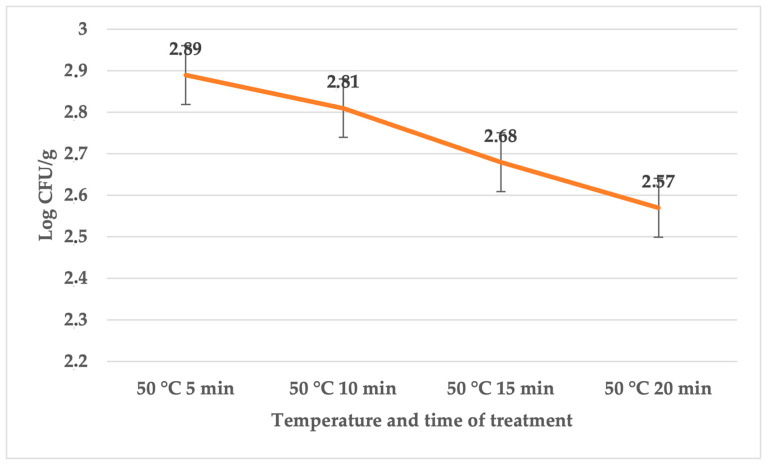
Number of *S. enterica* (log CFU/g) on day 1 in group treated with sage EO.

The primary challenge associated with sous vide procedures at lower temperatures is insufficient heat treatment. In our investigation, the initial day was dedicated to assessing the total bacterial count in the control, sage EO, and *S. enterica*-treated groups. The overall bacterial count ranged between 2.15 ± 0.006 (for treatment at 65 °C for 10 min) and 3.76 ± 0.08 log CFU/g (for treatment at 50 °C for 5 min) in the control groups. Meanwhile, in the treated groups, the bacterial count varied between 1.02 ± 0.02 (for treatment at 65 °C for 10 min) and 3.27 ± 0.12 log CFU/g (for treatment at 50 °C for 5 min) (Table 1). During a longer time at a temperature of 65 °C, the numbers were already zero. The coliforms bacteria were zero in all groups. The number of *Salmonella* counts (Figure 1) was only found in the control groups at 50 °C.

**Figure 2:**
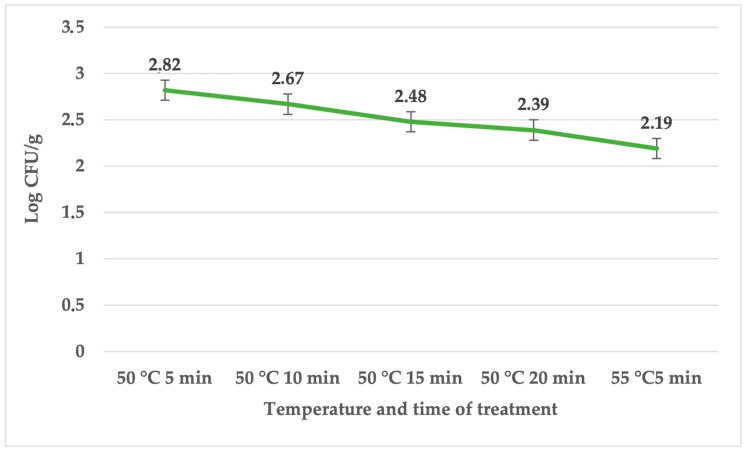
Number of *S. enterica* (log CFU/g) on day 3 in the group treated with sage EO.

Table 2 displays the impact of sage EO for the tested temperature treatments on the sous vide beef samples on day 3. The table presents the mean counts recorded in the samples with or without sage essential oil (EO) across different time points and under various heat treatments. Notably, the samples subjected to heat treatment at 50 °C for 5 min exhibited an elevated total bacterial count. In the control groups, the total number of bacteria ranged between 3.63 ± 0.06 and 4.41 ± 0.14 log CFU/g. Coliforms bacteria were only in the groups under the temperature of 50 °C and ranged between 2.49 ± 0.06 and 3.35 ± 0.03 log CFU/g (Table 3). The group treated with sage EO and *S. enterica* ranged between 2.08 ± 0.03 and 4.52 ± 0.06 log CFU/g. The *Salmonella* counts ranged between 2.19 ± 0.02 and 2.82 ± 0.06 log CFU/g (Figure 2).

**Figure 3:**
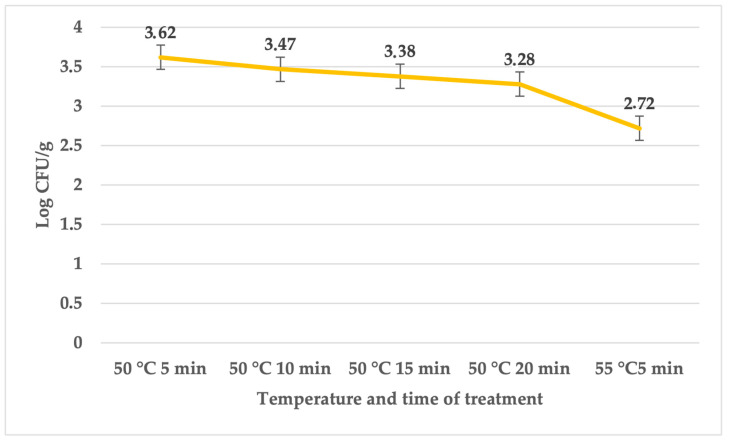
Quantity of *S. enterica* (expressed in log CFU/g) on the sixth day in the group subjected to treatment with sage essential oil.

The total bacterial count, as indicated in Table 4, ranged between 4.24 ± 0.06 (for treatment at 60 °C for 20 min) and 5.20 ± 0.04 log CFU/g in the control groups. In contrast, the treated groups exhibited counts between 2.66 ± 0.03 (for treatment at 60 °C for 20 min) and 4.70 ± 0.06 log CFU/g (for treatment at 50 °C for 5 min). On the sixth day, the coliform bacteria quantity (refer to Table 5) fluctuated between 2.19 ± 0.02 (for treatment at 60 °C for 20 min) and 2.9 ± 0.07 log CFU/g in the control groups, while in the treated groups, it ranged between 3.22 ± 0.06 log CFU/g (for treatment at 50 °C for 20 min) and 3.47 ± 0.07 log CFU/g (for treatment at 50 °C for 5 min). The count of *S. enterica* in the treated groups varied between 2.72 ± 0.05 and 3.62 ± 0.05 log CFU/g (refer to Figure 3).

### 3.2. Isolated Bacteria from Beef Meat

**Figure 4:**
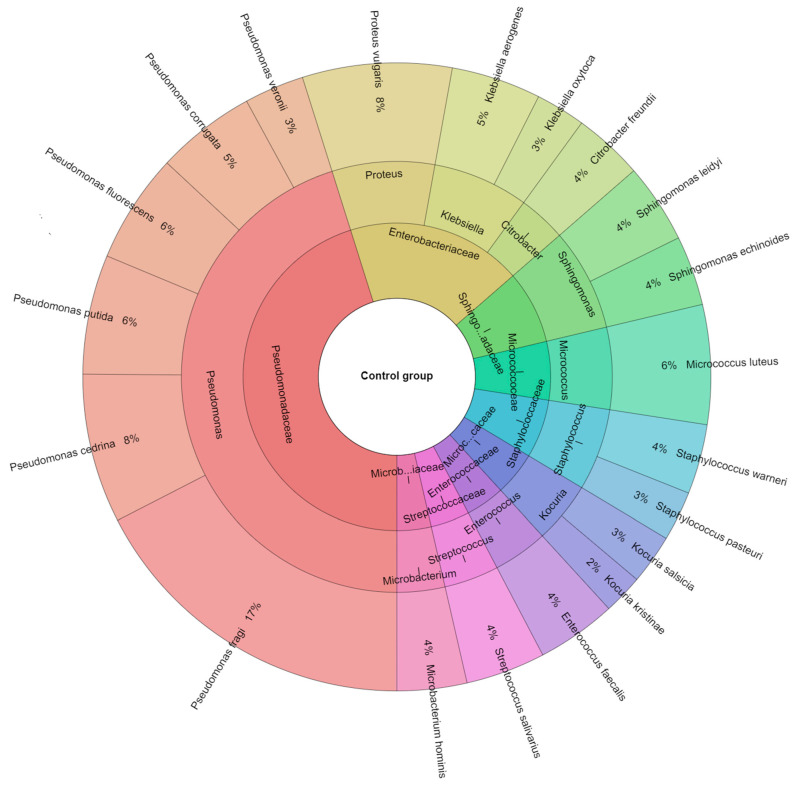
Krona chart depicting the species of bacteria isolated from the control groups.

**Figure 5:**
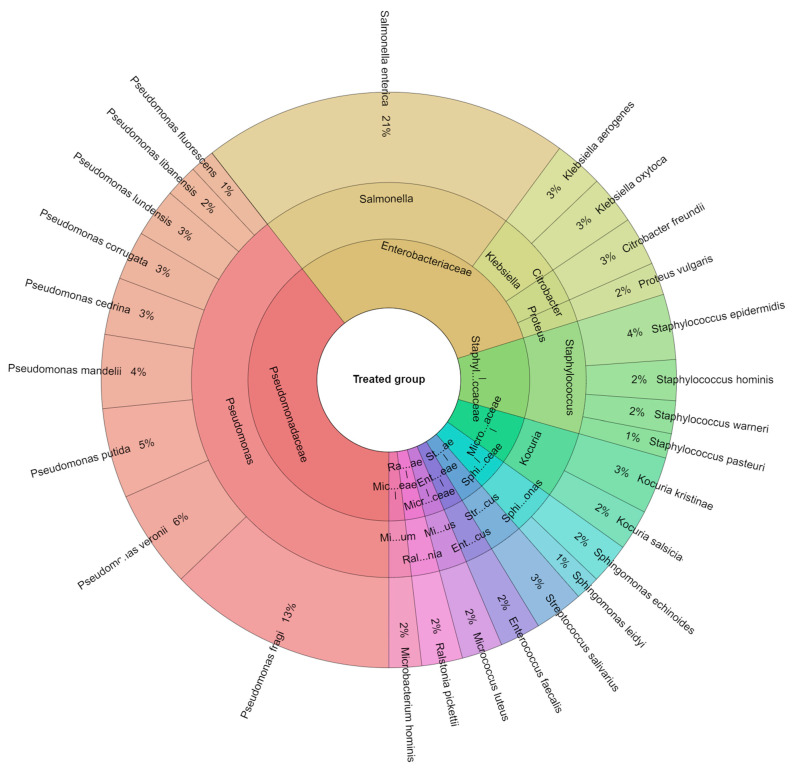
Krona chart illustrating the species of bacteria isolated from the treated groups.

A total of 413 isolates were identified from the sous vide beef meat samples in both the control group and treated groups. Figure 4 illustrates that 8 families, 14 genera, and 20 species were isolated from the control group. In this investigation, *Pseudomonas fragi* (17%), *Proteus vulgaris* (8%), and *Pseudomonas cedrina* (8%) were the most isolated species. The sous vide beef meat treatment group contained 9 families, 12 genera, and 27 species (Figure 5); in this group, the most isolated species (21%) was *S. enterica*. On the other hand, *Pseudomonas fragi* (13%), *Pseudomonas veronii* (6%), and *Pseudomonas putida* (5%) were the other bacteria species most frequently isolated from the treated group.

## 4. Discussion

Both consumers and food processors have indicated a wish to employ fewer synthetic chemicals to preserve food. Many ailments and conditions can benefit from using medicinal plants as a potential option [16,17,18,19,20]. Extracts and EOs from common culinary herbs, spices, and aromatic plants that exhibit pronounced antibacterial action have recently attracted much attention. According to Marino et al. [21], such compounds can stop or slow the growth of bacteria that produce toxins and/or pathogens in food. In our research, the antimicrobial effect of sous vide and sage EO was evaluated against *S. enterica*. The microbiological safety of foods continues to be a top concern for consumers, regulatory bodies, and the food industry globally, despite the variety of preservation strategies available. In recent years, numerous large-scale outbreaks of *Salmonella* have been a significant cause of food poisoning globally [22]. Antibiotics are a key tactic for eliminating these bacteria, and they are frequently used therapeutically and preventatively to treat and prevent salmonellosis in humans and animals. However, the use of antibiotics inevitably leads to the development of drug resistance, and recent research has revealed an increase in the incidence of antibiotic-resistant Salmonella in both humans and animals [23]. New, effective, and secure treatments for salmonellosis are thus required.

Sage has been valued as a spice from the beginning of time. As is the case today, ancient Egyptians, Greeks, and Romans used this plant extensively in their cuisine. To make meat products last longer, sage was included in fresh cuisine. Sage leaves are also utilized as a flavoring ingredient in many Mediterranean cuisines today. Fresh or dried *Salvia officinalis* leaves are used as a seasoning, garnish, or appetizer in Italian soups, meat, chicken dishes, pasta, and potatoes. Sage leaves added to sliced potatoes help them to achieve a crispy finish when baked. People often drink sage tea made from fresh leaves, which is thought to help in healing stomach issues. It has also been reported that sage added to vinegar helps with conditions including diabetes, hormonal issues [24], stomach aches, flushing, depression [25], and excessive sweating [26].

Sous vide cooking is still quite popular for preparing various food products, including meat. The examination of low-temperature sous vide cooking is the result of growing concern over the incorrect application of this cooking technique. In this experiment, sous vide cooking effectively reduced the total count of bacteria, coliform bacteria, and *Salmonella* present. *Salmonella*-inoculated chicken breasts were sous vide cooked, and the D-values at 55 °C for control samples were 47.65 ± 3.68 min and 34.12 ± 1.73 min for samples marinated in acidic teriyaki sauce [27]. For ground beef samples inoculated with *E. coli* O157:H7, sous vide was able to produce a D-value of 67.79 ± 5.48 min [28], and a D-value of 33.62 min [29] was achieved in samples inoculated with *L. monocytogenes*. After being vacuum-packed and injected with *L. monocytogenes*, the samples were cooked sous vide for the prescribed time at temperatures of 50, 55, 60, and 65 °C. The amount *of L. monocytogenes*, the total bacterial count, and the number of coliform bacteria were measured in control and treated groups of sous vide beef tenderloin on days 0, 3, 6, 9, and 12. The *L. monocytogenes*, coliform bacteria, and total bacterial count all increased. On each day that was measured, the samples belonging to the test group that had been exposed to a temperature of 50 °C for 5 min showed a higher total bacterial count [14].

Game meat samples inoculated with *L. monocytogenes* produced D-values at 50 °C of 100.2 ± 13.3 min for wild boar and 49.2 ± 2.0 min for roe deer [30], demonstrating that goods can be safely cooked at temperatures lower than 54.4 °C. The significant variation in D-values between the wild boar and roe deer employed in this study again emphasizes the significance of product validation. Holding temperature and time combinations are insufficient to obtain the requisite microbial lethality safety, and there is also a danger of residual thermo-tolerant microorganisms growing. Studies [31,32,33,34,35] show that spore germination can occur in sous vide cooked meat samples. Nevertheless, these experiments applied high-temperature heat treatments (62 °C–100 °C), extended refrigeration, and/or temperature abuse storage over several days and weeks.

To increase the safety of the meat product during storage, Šojic et al. [36] evaluated the efficacy of *S. officinalis* herbal dust (a by-product of the food industry) essential oil (0.05–0.1 L/g) against microbial development in fresh pork sausages. At 0.05 L/g, adding this essential oil decreased the microbiological growth in fresh pig sausages while having no adverse effects on the meat product’s sensory qualities.

It is a global issue that an increasing number of germs are resistant to antibiotics and are more tolerant of the current preservation procedures. Food processors and consumers are becoming increasingly interested in switching from synthetic preservatives to natural plant-derived antimicrobial preservatives to preserve food [37,38,39,40,41]. It has been demonstrated that the antibacterial properties of herbs, spices, and their essential oils exert antimicrobial activity against food spoilage and microorganisms present in food [42,43]. *Salvia* plant’s antibacterial properties may impact the incidence of vulnerable and resistant foodborne pathogens. Thus, essential oils and extracts have a potential to be used as alternatives to the growing usage of synthetic preservatives to improve microbiological food safety. According to the studies by Abdelkader et al. [44] and Miladinovic [45], *S. officinalis* essential oil has been shown to have antibacterial activity against *Escherichia coli*, *Salmonella enteritidis*, *Bacillus cereus*, *Bacillus subtilis*, *Candida albicans*, *Staphylococcus aureus*, and *Aspergillus niger*.

*Pseudomonas fragi* was the most prevalently isolated species in our study in both groups, with the exception *S. enterica* inoculated in the treated groups. In the investigation conducted by Gál et al. [15], the most frequently isolated species were *Kocuria salcida*, *Pantotea agglomerans*, *Hafnia alvei*, and *Pseudomonas fragi*. In the treated groups, *P. fragi*, *Lysinibacillus xylanitaticus*, *H. alvei*, and *Pseudomonas graminis* were the other bacterial species most commonly identified. The raw meat microbiota composition corresponds to previous papers on the bacterial genera found in raw beef and the equipment used for preparing beef cuts [46,47,48,49].

## 5. Conclusions

This study tested the antimicrobial effects of *Salvia officinalis* essential oil combined with the sous vide technique against foodborne pathogens belonging to *Salmonella enterica* in beef tenderloin. Intact beef samples that were inoculated with *Salmonella enterica* were safely reheated using sous vide cooking at 50, 55, 60, and 65 °C for 5, 10, 15, and 20 min. The *Salmonella enterica* levels, total bacterial counts, and coliform counts in the beef kept at 50 °C for 5, 10, 15, and 20 min did not fall to levels that could be considered safe. In our work, with the increase in temperature and time, the number of unique microorganisms decreased. The results of our experiments revealed that, on one hand, the combination of a higher temperature and longer time can reduce the number of microorganisms, and on the other hand, the application of plant essential oil inhibited the addition of *S. enterica* bacteria. Essential oils have shown very strong potential in extending the shelf life of food, as was also proven in our results. To consider this product safe to eat, an additional step of heat killing or cooking at a higher temperature using the sous vide method must be used. The combined sage EO with sous vide treatment is a good alternative for storing beef samples at 4 ± 2 °C.

## Figures and Tables

**Table 1:** The outcomes of the bacterial total count in the control groups and the groups subjected to sage EO and *S. enterica* treatments (expressed in log CFU/g) on the first day.

Treatment	Temperature (°C)	Time (min)	Average	SD	*p* Value
BM	50	5	3.76	0.08	4.114 × 10^−3^
BMSEEO	50	5	3.27	0.12	
BM	50	10	3.63	0.17	2.110 × 10^−3^
BMSEEO	50	10	2.90	0.06	
BM	50	15	3.35	0.09	3.302 × 10^−4^
BMSEEO	50	15	2.72	0.04	
BM	50	20	3.35	0.05	3.219 × 10^−2^
BMSEEO	50	20	2.58	0.05	
BM	55	5	3.28	0.05	1.814 × 10^−2^
BMSEEO	55	5	2.47	0.05	
BM	55	10	3.16	0.02	1.315 × 10^−2^
BMSEEO	55	10	2.22	0.05	
BM	55	15	3.08	0.04	1.230 × 10^−2^
BMSEEO	55	15	2.15	0.03	
BM	55	20	2.94	0.05	8.874 × 10^−3^
BMSEEO	55	20	1.94	0.05	
BM	60	5	2.68	0.07	5.179 × 10^−2^
BMSEEO	60	5	1.68	0.07	
BM	60	10	2.62	0.04	2.366 × 10^−2^
BMSEEO	60	10	1.62	0.04	
BM	60	15	2.35	0.06	1.631 × 10^−2^
BMSEEO	60	15	1.35	0.06	
BM	60	20	2.17	0.03	7.026 × 10^−3^
BMSEEO	60	20	1.17	0.03	
BM	65	10	2.03	0.006	1.527 × 10^−3^
BMSEEO	65	10	1.02	0.02	

BM: Fresh beef meat vacuum-sealed in polyethylene (PE) bags, stored anaerobically at 4 ± 2 °C, and treated at temperatures ranging between 50 and 65 °C for durations of 5 to 20 min. BMSEEO: Fresh beef meat treated with *S. enterica* and 2% sage essential oil (EO), vacuum-sealed into PE bags, stored anaerobically at 4 ± 2 °C, and treated at temperatures ranging between 50 and 65 °C for durations of 5 to 20 min.

**Table 2:** The outcomes of the bacterial total count in the control groups and the groups subjected to sage EO and *S. enterica* treatments (expressed in log CFU/g) on the third day.

Treatment	Temperature (°C)	Time (min)	Average	SD	*p* Value
BM	50	5	4.41	0.14	3.003 × 10^−1^ *
BMSEEO	50	5	4.52	0.06	
BM	50	10	4.36	0.05	4.214 × 10^−2^
BMSEEO	50	10	4.45	0.03	
BM	50	15	4.28	0.04	3.377 × 10^−2^
BMSEEO	50	15	4.40	0.06	
BM	50	20	4.17	0.04	2.279 × 10^−2^
BMSEEO	50	20	4.44	0.12	
BM	55	5	4.08	0.03	5.406 × 10^−4^
BMSEEO	55	5	4.31	0.02	
BM	55	10	3.95	0.03	1.253 × 10^−4^
BMSEEO	55	10	2.50	0.17	
BM	55	15	3.79	0.12	1.413 × 10^−2^
BMSEEO	55	15	2.16	0.01	
BM	55	20	3.63	0.06	7.191 × 10^−3^
BMSEEO	55	20	2.08	0.03	

BM: fresh beef meat vacuum-sealed in polyethylene (PE) bags, stored anaerobically at 4 ± 2 °C, and treated at temperatures ranging between 50 and 55 °C for durations of 5 to 20 min. BMSEEO: Fresh beef meat treated with *S. enterica* and 2% sage essential oil (EO), vacuum-sealed into PE bags, stored anaerobically at 4 ± 2 °C, and treated at temperatures ranging between 50 and 55 °C for durations of 5 to 20 min. * The provided data did not exhibit statistical significance at the 95% confidence level.

**Table 3:** Coliform bacterial counts (expressed in log CFU/g) in the groups subjected to treatments with sage essential oil (EO) and *S. enterica*, as well as in the control groups, were assessed on the third day.

Treatment	Temperature (°C)	Time (min)	Average	SD	*p* Value
BM	50	5	2.81	0.05	5.500 × 10^−2^
BMSEEO	50	5	3.35	0.03	
BM	50	10	2.49	0.06	2.833 × 10^−2^
BMSEEO	50	10	3.23	0.02	

BM: fresh beef meat vacuum-sealed in polyethylene (PE) bags, stored anaerobically at 4 ± 2 °C, and treated at 50 °C for durations ranging between 5 and 10 min. BMSEEO: Fresh beef meat treated with *S. enterica* and 2% sage essential oil (EO), vacuum-sealed into PE bags, stored anaerobically at 4 ± 2 °C, and treated at 50 °C for durations ranging between 5 and 10 min.

**Table 4:** The outcomes of the bacterial total count in the control groups and the groups subjected to sage EO and *S. enterica* treatments (expressed in log CFU/g) on the sixth day.

Treatment	Temperature (°C)	Time (min)	Average	SD	*p* Value
BM	50	5	5.20	0.04	2.519 × 10^−4^
BMSEEO	50	5	4.70	0.06	
BM	50	10	5.08	0.01	1.213 × 10^−4^
BMSEEO	50	10	4.59	0.06	
BM	50	15	4.93	0.06	5.822 × 10^−4^
BMSEEO	50	15	4.57	0.03	
BM	50	20	4.83	0.04	1.144 × 10^−2^
BMSEEO	50	20	4.54	0.11	
BM	55	5	4.72	0.04	3.776 × 10^−4^
BMSEEO	55	5	4.42	0.03	
BM	55	10	4.52	0.06	1.357 × 10^−4^
BMSEEO	55	10	3.55	0.10	
BM	55	15	4.36	0.04	1.004 × 10^−4^
BMSEEO	55	15	3.25	0.12	
BM	55	20	4.24	0.06	5.148 × 10^−3^
BMSEEO	55	20	2.66	0.03	

BM: fresh beef meat vacuum-sealed in polyethylene (PE) bags, stored anaerobically at 4 ± 2 °C, and treated at temperatures ranging between 50 and 55 °C for durations of 5 to 20 min. BMSEEO: Fresh beef meat treated with *S. enterica* and 2% sage essential oil (EO), vacuum-sealed into PE bags, stored anaerobically at 4 ± 2 °C, and treated at temperatures ranging between 50 and 55 °C for durations of 5 to 20 min.

**Table 5:** Coliform bacterial counts in the groups subjected to treatments with sage essential oil (EO) and *S. enterica*, as well as in the control groups, were assessed on the sixth day.

Treatment	Temperature (°C)	Time (min)	Average	SD	*p* Value
BM	50	5	2.90	0.07	4.896 × 10^−4^
BMSEEO	50	5	3.47	0.07	
BM	50	10	2.67	0.10	3.394 × 10^−4^
BMSEEO	50	10	3.37	0.05	
BM	50	15	2.37	0.05	1.369 × 10^−2^
BMSEEO	50	15	3.28	0.04	
BM	50	20	2.19	0.02	1.707 × 10^−2^
BMSEEO	50	20	3.22	0.06	

BM: Fresh beef meat vacuum-sealed in polyethylene (PE) bags, stored anaerobically at 4 ± 2 °C, and treated at 50 °C for durations ranging between 5 and 20 min. BMSEEO: Fresh beef meat treated with *S. enterica* and 2% sage essential oil (EO), vacuum-sealed into PE bags, stored anaerobically at 4 ± 2 °C, and treated at 50 °C for durations ranging between 5 and 20 min.

## Data Availability

The data presented in this study are available on request from the corresponding author.
